# Monitoring telehealth vomiting calls as a potential public health early warning system for seasonal norovirus activity in Ontario, Canada

**DOI:** 10.1017/S0950268818003357

**Published:** 2019-03-08

**Authors:** S. L. Hughes, R. A. Morbey, A. J. Elliot, S. A. McEwen, A. L. Greer, I. Young, A. Papadopoulos

**Affiliations:** 1 Department of Population Medicine, University of Guelph, Guelph, Ontario, Canada; 2 Real-time Syndromic Surveillance Team, Field Service, National Infection Service, Public Health England, Birmingham, UK; 3 School of Occupational and Public Health, Ryerson University, Toronto, Ontario, Canada

**Keywords:** Surveillance system, Norwalk agent and related viruses, gastroenteritis, surveillance

## Abstract

Norovirus is a predominant cause of infectious gastroenteritis in countries worldwide [1–5]. It accounts for approximately 50% of acute gastroenteritis (AGE) and >90% of viral gastroenteritis outbreaks [6, 7]. The incubation period ranges between 10 and 48 h and illness duration is generally 1–3 days with self-limiting symptoms; however, this duration is often longer (e.g. 4–6 days) in vulnerable populations such as hospital patients or young children [2, 8]. Symptomatic infection of norovirus presents as acute vomiting, diarrhoea, abdominal cramps and nausea, with severe vomiting and diarrhoea (non-bloody) being most common [2, 5, 9].

## Introduction

Norovirus is a predominant cause of infectious gastroenteritis in countries worldwide [1–5]. It accounts for approximately 50% of acute gastroenteritis (AGE) and >90% of viral gastroenteritis outbreaks [6, 7]. The incubation period ranges between 10 and 48 h and illness duration is generally 1–3 days with self-limiting symptoms; however, this duration is often longer (e.g. 4–6 days) in vulnerable populations such as hospital patients or young children [2, 8]. Symptomatic infection of norovirus presents as acute vomiting, diarrhoea, abdominal cramps and nausea, with severe vomiting and diarrhoea (non-bloody) being most common [2, 5, 9].

Norovirus activity occurs with predictable seasonality. Although the specific months vary, activity peaks during cold weather winter months in temperate climates, similar to other common respiratory viruses (e.g. influenza and rhinovirus) [2, 10]. Due to its predictable winter seasonality, norovirus is often referred to as ‘winter vomiting disease’ [11, 12]. However, outbreaks can occur throughout the year, especially in years when a new norovirus variant has emerged [13].

Traditional surveillance of norovirus activity plays an essential role in the identification of seasonal trends, disease burden in the population and the extent of geographical spread. This traditional surveillance is reliant upon datasets such as laboratory submissions and outbreak case reporting to carry out these tasks. However, due to the self-limiting nature and short duration of norovirus illness, public health surveillance is limited by under-reporting and subsequent time lags [14–17]. Hall *et al*. demonstrated that only 15% of individuals affected with AGE illness seek medical intervention, and of those, diagnostic testing samples were only requested from 13% [14]. Other studies have described the absence of routine surveillance and frequent under-reporting, which affect the overall ability to monitor disease activity [15, 18]. The time lags associated with traditional laboratory surveillance methods have long been recognised as a limitation for disease surveillance due to their delayed effects on public health interventions. A study performed by Ashford *et al*. demonstrated across a number of public health scenarios that traditional reporting techniques had a delay of 0–26 days between the index case and when the problem was identified through surveillance [17]. Such lags include the time taken for general practitioners to submit samples to the laboratory, and for the specific laboratory tests to be performed and reported [17]. These delays reduce the timeliness of disease monitoring and affect the ability to implement effective public health interventions.

This paper presents a proposal for an early warning syndromic surveillance system using telehealth syndromic surveillance call data to detect winter norovirus activity in Ontario, Canada. The use of real-time surveillance for monitoring norovirus activity could greatly improve the ability of public health authorities to monitor illness, identify seasonal increases and thus implement timelier interventions to reduce subsequent impact.

## Methods

This study was conducted using data from the province of Ontario, Canada. It is the largest province in the country with a population of roughly 14.1 million residents [19].

### Syndromic surveillance telehealth call data

Telehealth Ontario call data were obtained from the Ontario Ministry of Health and Long-Term Care and Sykes Assistance Services Corporation for the period 17 June 2011–29 August 2015. Telehealth Ontario is a nurse-led service available to all residents of Ontario, 24 h a day, 7 days a week. The system provides Ontarians with immediate health information and advice for a variety of medical concerns, and where appropriate provides triage using Schmitt–Thompson guidelines to direct them to the most appropriate health care service, e.g. a general practitioner appointment or an immediate visit to an emergency department [20, 21]. It is estimated that Telehealth Ontario receives approximately 75–80 000 calls per month [22, 23]. The telehealth data were received as daily call counts and aggregated into weekly counts for sufficient sample sizes in analyses. All AGE calls were requested which included the chief complaints: vomiting, diarrhoea, vomiting and diarrhoea, vomiting without diarrhoea (subsequently coded as ‘vomiting’), abdominal pain and vomiting with blood. For the purposes of this study, the ‘vomiting’, ‘diarrhoea’ and ‘vomiting and diarrhoea’ chief complaints were included in analyses (subsequently referred to as the ‘relevant chief complaints’).

### Norovirus laboratory reports

Norovirus laboratory data were obtained from Public Health Ontario for the period 17 June 2011–30 September 2014. Laboratory data were defined as stool samples obtained from residents of Ontario with suspected norovirus, which were submitted to Public Health Ontario by a physician for confirmatory testing as part of routine patient management protocols. Samples were tested for a variety of gastrointestinal (GI) pathogens using polymerase chain reaction, electron microscopy or immunochromatographic testing; the presence or absence of norovirus in the sample was reported, however no genotype information was available. The data were received as daily norovirus test results and aggregated into weekly counts to enable sufficient sample sizes in analyses, reduce the impact of under-reporting during weekends and holidays, and to account for the availability of laboratory data which are often available on a weekly basis. For the purposes of this study, the collection dates (the date of sample collection from infected patients), and only samples which tested positive for the presence of norovirus were utilised in analyses.

### Negative binomial regression analyses

Negative binomial regression analyses were performed to compare the telehealth and laboratory datasets. The telehealth data were used as the outcome variable and the laboratory data as a predictor. Negative binomial regressions were selected due to the fact count data were analysed.

Both the telehealth and laboratory datasets were analysed over the period 17 June 2011–30 September 2014 (week 24, 2011–week 4 02 014). A ‘public holiday’ variable was added to both datasets to indicate the occurrence of an Ontario-wide statutory holiday; this was included because previous studies have shown the effects public holidays have on call volumes and the necessity to treat holidays differently than normal days [24]. A ‘date’ variable was also added to the datasets to indicate the start of the week analysed; this was included as a trend variable to assess long-term patterns in the data.

The laboratory dataset was used at time 0 and shifted 1, 2 and 3 weeks forward to construct lag variables (*t*_0_, *t*_1_, *t*_2_ and *t*_3_, respectively) to observe whether a statistically significant time lag existed between the datasets. Prior to performing this study, an assumption was made that the telehealth data might precede the laboratory data by a number of weeks [23, 25, 26].

Sets of four negative binomial regression models were constructed with the original laboratory data (*t*_0_), and the *t*_1_, *t*_2_ and *t*_3_ lag variables as predictor variables. These predictors were regressed against weekly counts of calls to telehealth using the following parameter combinations: all-ages and all relevant chief complaints (‘index parameter model’), all-ages and vomiting-only chief complaints (‘vomiting’ and ‘vomiting and diarrhoea’ calls only), 0–4 ages and all relevant chief complaints, ⩾65 ages and all relevant chief complaints, 0–4 ages and vomiting-only chief complaints, and ⩾65 ages and vomiting-only chief complaints. These various combinations were tested to observe whether the index parameter model was the most sensitive to early warning, or if a combination of indicators were most sensitive. The specific age groups selected were tested as they are known to be disproportionately affected by norovirus and other gastroenteritis infections [5, 9, 27], and the vomiting-only chief complaints were tested because vomiting is the primary symptom of norovirus.

Included in each of these models were the public holiday and date variables. The (McFadden's) pseudo-*R*^2^ values of these models were compared with each other to determine the best-fitting model; however, it should be noted that pseudo-*R*^2^ values should not be interpreted the same as *R*^2^ for normal data.

All negative binomial regression analyses were performed in Stata v.13.0 (StataCorp LLC, College Station, Texas, USA).

### Early warning system threshold calculations

The telehealth data were plotted from 19 June 2011 to 29 August 2015 (week 25, 2011–week 34, 2015). A downward trend in call volumes was observed during this time period; therefore, a linear trendline was inserted to calculate the overall slope. This slope (−0.46) was subsequently used to standardise the overall baseline of the data, setting the very first week as the referent.

The periods of increased laboratory reports of norovirus were observed to be regular, within weeks 44–24 (Oct/Nov–June) and were therefore defined as the active season; weeks 25–43 (June–Oct) were defined as out-of-season. Using these weeks as approximate measures for the winter norovirus season, the average weekly call volume for the out-of-season periods was calculated (referred to as the baseline). Using this baseline, the standard deviation (s.d.) was then calculated and modelled incrementally (up to 4s.d.) above the overall average for the out-of-season call volumes for the period of 19 June 2011 to 29 August 2015. This was done using Shewhart (control) chart method [28], a method which displays the mean of the statistic on a graph and models s.d. of the statistic above and/or below the mean to indicate a threshold of data variation.

All threshold calculations were executed using Microsoft Office (Excel) 2016.

## Results

During the 17 June 2011–29 August 2015 time period, a total of 184 249 calls were made to Telehealth Ontario regarding the relevant chief complaints. Additionally, during the 17 June 2011–30 September 2014 time span, 2166 norovirus-positive samples were submitted to Public Health Ontario for testing. There was an average of 841 calls to telehealth per week regarding the chief complaints, and an average of 12 norovirus-positive samples submitted for laboratory testing per week.

### Negative binomial regression analyses

**Fig. 1.**
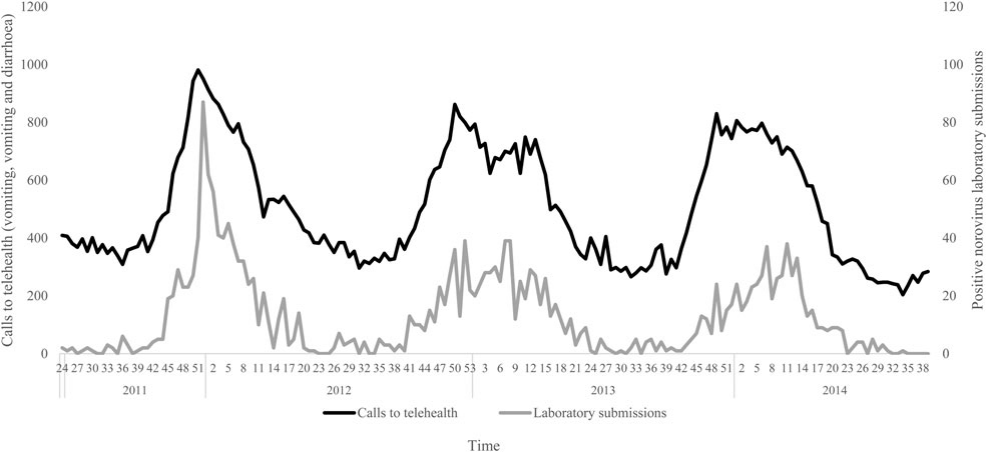
Weekly telehealth vomiting calls (‘vomiting’ and ‘vomiting and diarrhoea’ chief complaints) and laboratory-positive norovirus submissions, 17 June 2011–30 September 2014.

The negative binomial regression models demonstrated that the all-ages, vomiting-only chief complaint *t*_0_, *t*_1_, *t*_2_ and *t*_3_ models were the best-fitting of all the parameter combinations tested. This parameter combination produced the highest pseudo-*R*^2^ values when compared with all other parameter combinations (Table 1). In this combination, the *t*_0_ and *t*_1_ variables were not significant (*P* > 0.05); however, the *t*_2_ and *t*_3_ lag variables were significant (*P* = 0.004, 0.003, respectively) (Table 2). Although both the *t*_2_ and *t*_3_ lag variables were significant, the fit of the *t*_2_ model (pseudo-*R*^2^ = 0.1861) was higher than that of the *t*_3_ model (pseudo-*R*^2^ = 0.1852) (Table 1). A visual comparison/overlay of the telehealth and laboratory datasets over the timeframe used in the regression analyses (17 June 2011–30 September 2014) is displayed in Figure 1, and the full model results are described in Table 2.

**Table 1.** Pseudo-*R*^2^ values for all negative binomial regression models

Model parameters		Model type (stage of lag)^a^			
Age (years)	Telehealth chief complaints	*t*_0_	*t*_1_	*t*_2_	*t*_3_
**All**	**Includes vomiting**^b^	0.1733	0.1828	0.1861	0.1852
0–4	Includes vomiting	0.1760	0.1834	0.1849	0.1841
0–4	Includes vomiting and/or diarrhoea^c^	0.1663	0.1764	0.1783	0.1791
All	Includes vomiting and/or diarrhoea	0.1561	0.1702	0.1758	0.1748
⩾65	Includes vomiting and/or diarrhoea	0.0878	0.0845	0.0993	0.0831
⩾65	Includes vomiting	0.0779	0.0767	0.0899	0.0802

The highest value in each row is underlined. Bolded text indicates the favoured model deduced from analyses.

a Laboratory data used in negative binomial models was regressed at time 0 (*t*_0_), and shifted 1 (*t*_1_), 2 (*t*_2_) and 3 (*t*_3_) weeks in the future, separately against telehealth calls.

b Vomiting-only calls encompass ‘vomiting’ and ‘vomiting and diarrhoea’ telehealth chief complaints.

c Vomiting and diarrhoea calls encompass ‘vomiting’, ‘diarrhoea’ and ‘vomiting and diarrhoea’ telehealth chief complaints.

**Table 2.** Model results for norovirus laboratory data predictor variable in all negative binomial regression models

Model parameters		Model type (stage of lag)^a^											
		*t*_0_			*t*_1_			*t*_2_			*t*_3_		
Age (years)	Telehealth chief complaints	*P*-value	Coefficient	95% CI	*P*-value	Coefficient	95% CI	*P*-value	Coefficient	95% CI	*P*-value	Coefficient	95% CI
**All**	**Includes vomiting^b^**	0.442	−0.0010	(−0.0036) to 0.0016	0.964	0.000055	(−0.0023) to 0.0024	0.004	0.0030	0.00097–0.0051	0.003	0.0028	0.00093–0.0047
0–4	Includes vomiting	0.630	0.00070	(−0.0021) to 0.0035	0.804	0.00036	(−0.0025) to 0.0032	0.040	0.0027	0.00013–0.0052	0.039	0.0025	0.00013–0.0049
0–4	Includes vomiting and/or diarrhoea^c^	0.544	0.00080	(−0.0018) to 0.0034	0.306	0.0013	(−0.0012) to 0.0038	0.017	0.0027	0.00047–0.0049	0.002	0.0031	0.0011–0.0051
All	Includes vomiting and/or diarrhoea	0.738	0.00038	(−0.0019) to 0.0026	0.064	0.0018	(−0.00010) to 0.0037	<0.001	0.0034	0.0019–0.0050	<0.001	0.0031	0.0017–0.0046
⩾65	Includes vomiting and/or diarrhoea	<0.001	0.0082	0.0048–0.012	<0.001	0.0082	0.0047–0.012	<0.001	0.0096	0.0067–0.012	<0.001	0.0081	0.0052–0.011
⩾65	Includes vomiting	<0.001	0.0068	0.0042–0.0093	<0.001	0.0065	0.0041–0.0090	<0.001	0.0077	0.0056–0.0097	<0.001	0.0058	0.0036–0.0080

CI, confidence interval.

Significant *P*-values in all rows are underlined. Bolded text indicates the favoured model deduced from analyses.

a Laboratory data used in negative binomial models were regressed at time 0 (*t*_0_), and shifted 1 (*t*_1_), 2 (*t*_2_) and 3 (*t*_3_) weeks in the future, separately against telehealth calls.

b Vomiting-only calls encompass ‘vomiting’ and ‘vomiting and diarrhoea’ telehealth chief complaints.

c Vomiting and diarrhoea calls encompass ‘vomiting’, ‘diarrhoea’ and ‘vomiting and diarrhoea’ telehealth chief complaints.

The public holiday variable was not significant in any of the *t*_0_, *t*_1_, *t*_2_ and *t*_3_ all-ages, vomiting-only chief complaint models (*P* ⩾ 0.05). However, due to its practical significance and importance in syndromic models demonstrated by other research groups worldwide, it was kept in the final models. The date variable was observed to be significant in all of the *t*_0_, *t*_1_, *t*_2_ and *t*_3_ all-ages, vomiting-only chief complaint models (*P* < 0.05), and was therefore included in the final models.

### Early warning threshold calculations

**Fig. 2.**
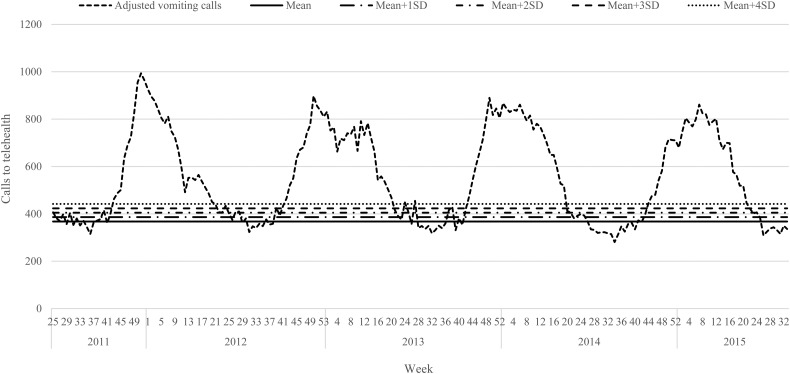
Weekly telehealth vomiting calls adjusted to remove long-term trend, with the ‘baseline’ mean number of calls and standard deviations marked, 19 June 2011–29 August 2015.

Trend-adjusted call volumes were plotted and visualised (Fig. 2). s.d. were calculated on the baseline (average number of weekly calls recorded during the out-of-season time period) of vomiting-only calls. Each s.d. level of vomiting-only calls was set as an early warning threshold across each of the four winter norovirus seasons analysed. Call volumes were observed noting the week during which each s.d. threshold was breached, and the peak week of activity during each season (Table 3). Where weekly vomiting call volumes breached the s.d. level of vomiting-only calls to telehealth for 3 consecutive weeks, this was noted as a winter norovirus season early warning alarm.

**Table 3.** Telehealth vomiting call volumes and threshold values, and start week for winter norovirus activity; values by standard deviation increment used for thresholds

	Mean + 2s.d. (404 calls)^a^		Mean + 3s.d. (423 calls)^a^		Mean + 4s.d. (441 calls)^a^	
Year	Week	# Calls	Week	# Calls	Week	# Calls
2011	44	462	44	462	44	462
2012	42	433	42	433	43	465
2013	43	419	44	474	44	474
2014	45	444	45	444	45	444

a Calculated threshold values (number of calls).

The presence of false alarms was assessed during the out-of-season weeks. Applying the recommended control chart standard of using the mean + 3s.d. to devise an early warning threshold [28], five false alarms would have been triggered during the 19 June 2011–29 August 2015 period assessed. Alarms were declared as false if they surpassed the 3s.d. level of vomiting-only calls, but not for 3 consecutive weeks. There were two occasions in 2012 (week 25, 433 vomiting-only calls; week 40, 426 vomiting-only calls), and three occasions in 2013 (week 25, 448 vomiting-only calls; week 28, 454 vomiting-only calls; week 39, 430 vomiting-only calls) that surpassed the 3s.d. level of vomiting-only calls. This is in comparison to the eight false alarms that would have been triggered using the 2s.d. level of vomiting-only calls, which has also been recommended by other researchers [29].

## Discussion

### Main findings

This study provides an initial validation that there is a statistical association between vomiting calls made to a telehealth system in Ontario and norovirus-positive laboratory test results. Regression analysis provided evidence of a 2-week lag of telehealth vomiting calls ahead of norovirus-positive laboratory reports, thus revealing potential to provide early warning of seasonal norovirus activity by monitoring telehealth vomiting calls. A series of early warning thresholds were developed based upon previously published methods and tested for the triggering of alerts in the study period.

Telehealth calls with vomiting as the chief complaint in all ages demonstrated the timeliest prediction of the winter norovirus season, thus were the focus of this analysis. This timely indication over all other telehealth parameters assessed is likely due to vomiting being the most predominant symptom of the illness in adults [5, 9]. These results were comparable to those found by Loveridge *et al*., who demonstrated that vomiting-only calls in the <5 age group gave the most sensitive prediction of the winter norovirus season [25]. A significant 2-week prediction of telehealth calls ahead of norovirus laboratory reports was also observed, emphasizing the value of using community-based surveillance systems that monitor initial presentation of symptoms at a population level, rather than subsequent patient-level laboratory reports. This also highlights the importance of integrating syndromic surveillance systems with traditional systems for enhanced disease surveillance. Loveridge *et al*. found an early warning of 6–11 weeks in the <5 ages; however, were able to calculate AGE calls as a proportion of total calls to telehealth and adjust the system to the most sensitive indicators [25].

A set of winter norovirus early warning alarm thresholds were devised for the four winter periods analysed, indicating the week during which an early warning alert would be appropriate. Following analyses, the recommended control chart standard of the mean + 3s.d. was found to be ideal for this system. This is because analyses demonstrated that the 3s.d. level would have only triggered five false alarms and would not have significantly delayed public health action to mitigate winter norovirus activity. Selecting a threshold with a shorter time delay would have incorporated more false alarms (2s.d. level), and a threshold with fewer false alarms would have delayed public health action (4s.d. level). Therefore, this recommendation was most appropriate at capturing the winter norovirus season without setting off a high number of false alerts in the out-of-season periods, and without significant time delay. This 3s.d. level agrees with several studies in the fields of syndromic surveillance and/or norovirus, which have followed this metric to accurately identify variation within sample statistics [28, 30–32]. However, using the 2s.d. in addition to the 3s.d. threshold could also prove to be useful as a possible additional early alert signal to warn public health authorities that an alarm may be triggered the following week. This would allow earlier public health action to be considered even before an official alert is triggered.

While this telehealth early warning system demonstrated the strongest association with a 2-week prediction of the norovirus laboratory reports, additional time lags must be considered. The average time delay between sample collection and laboratory reporting was 2.6 days (Hughes *et al*., unpublished data). However, it is important to note that this was based upon analysis of retrospective data; prospectively, these data would not necessarily be tested or reported in a timely manner, with results often batched and/or reported at a later date, extending the delay to reporting. Therefore, prospective reporting of laboratory data always needs to incorporate these additional reporting delays, and further highlights the importance of having real-time data available for use in surveillance systems to detect disease activity as early as possible.

The observed decrease in the telehealth weekly vomiting calls was evident over the entire period analysed in this study. Such decreases may be due to the overall decrease in telehealth utilisation in Ontario and increased usage of the Internet for at-home health care advice. A high percentage of ill patients in countries worldwide are now utilizing the Internet as their first point of medical care [33, 34]; a study performed by the Pew Research Center in 2012 found that 31% of all cell phone owners utilised their devices for health-related advice (in comparison to 17% in 2010) by means of Internet searches, texting services and/or health applications [35]. These statistics, in combination with the decreases observed in telehealth call numbers as far back as 2009, may explain the general downward trend observed in the data in this study. To verify whether this decrease was limited just to the gastroenteritis reasons for call, or affected all telehealth calls, additional calculations comparing both the telehealth gastroenteritis call volumes and total call volumes (all reasons for calls) during the same timeframe would be required. However, these data were not available for this study.

### Strengths and limitations

This study successfully utilised two existing datasets to provide an understanding of seasonal norovirus activity in the community. The advantage of using telehealth data was the improved representativeness of community activity. Laboratory data are frequently under-representative of disease burden and are biased, with sampling predominantly occurring in patients from higher risk or vulnerable groups and those from institutionalised outbreaks, particularly from hospital or long-term care settings [23, 36]. Telehealth Ontario provides a service to all Ontarians, and better captures those patients who do not present to other healthcare services. In addition, this study was able to highlight that telehealth data were able to provide early warning in comparison with the laboratory data.

There were several limitations associated with the datasets used in this study. The underlying reasons why physicians requested stool samples, or what pathogens/tests were requested were unknown; these decisions were made as part of the routine patient care pathway, but not made available as part of the study dataset. This may have had some effect on the validity of the data. Furthermore, it was only possible to undertake a comparative analysis across three norovirus seasons due to a lack of data from 2015 in the laboratory dataset. The lack of sufficient daily data in both datasets prevented analysis at a more granular daily level, as laboratory submissions and telehealth call counts were aggregated to weekly counts due to low daily counts. Additionally, the overall decreasing trend of weekly vomiting calls to telehealth over the study period made the early warning threshold calculations challenging. Adding a slope adjustment to calculate an adjusted baseline was required; however, if adopted as a prospective system, further decreasing call volumes would need to be continually monitored and thresholds adjusted accordingly. Finally, the telehealth data monitored the presentation of patients’ reported symptoms and were not a confirmation of norovirus illness. The laboratory data were used to determine the sensitivity of vomiting calls to norovirus activity; however, it is still important to note that the telehealth data can only be used as a proxy for norovirus activity and will also be affected by seasonal increases in a range of other GI pathogens.

### Implications for public health practice

It is recommended that the early warning system proposed in this study be further validated with subsequent implementation and routine use in the province of Ontario, Canada. Adopting syndromic surveillance alongside existing laboratory surveillance would improve monitoring of disease and allow for timelier tracking of norovirus activity. This work also highlights the utility of telehealth call data to detect the onset of the winter norovirus season as early as possible, thus potentially facilitating health care professionals to mitigate the impact by implementing infection control measures, such as increased hand hygiene and patient isolation. Ward closures may also be initiated in high-risk settings (e.g. hospitals, long-term care homes) following the breaching of early warning thresholds [37]. Additionally, the development of an early warning system can also be implemented out-of-season to warn of potential unusual activity, which in the case of norovirus may be caused by the introduction of new genotype variants [13, 38].

### Future directions

If adopted into routine public health practice, the norovirus surveillance and early warning system developed in this study should be updated prospectively with daily data, should sufficient daily counts be available in Ontario, to provide the timeliest surveillance information. In addition, as telehealth data become more commonly utilised in public health practice, it will become more feasible to develop similar, well-adapted telehealth syndromic surveillance systems for the detection of a wider range of public health issues, including other communicable diseases (e.g. influenza) and environmental impacts (e.g. heatwaves). These systems could be adopted by other provinces across Canada which employ similar telehealth systems, such as the Tele-Care telehealth service in New Brunswick, and Health Link in Alberta [39, 40]. Both of these examples offer 24 h-a-day, 7-days-a-week nurse triaging services, similar to Telehealth Ontario, and could therefore be utilised in a provincial telehealth syndromic surveillance system.

Additional sources of syndromic data could be used to complement telehealth data, such as pharmacy purchases, Internet search queries, school absenteeism and general practitioner diagnoses, and integrated into existing laboratory and outbreak surveillance methods [26, 41]. In doing so, data gaps and delays would be reduced and more effective public health actions performed to protect Ontarians.

## References

[1] Morton VK, Thomas MK and McEwen SA (2015) Estimated hospitalizations attributed to norovirus and rotavirus infection in Canada, 2006–2010. Epidemiology and Infection 143, 3528–3537. 25991407 10.1017/S0950268815000734 PMC4657031

[2] Patel MM (2009) Noroviruses: a comprehensive review. Journal of Clinical Virology 44, 1–8. 19084472 10.1016/j.jcv.2008.10.009

[3] Yen C and Hall AJ (2013) Challenges to estimating norovirus disease burden. Journal of the Pediatric Infectious Diseases Society 2, 61–62. 26619443 10.1093/jpids/pis134 PMC5712442

[4] Widdowson MA (2005) Norovirus and foodborne disease, United States, 1991–2000. Emerging Infectious Diseases 11, 95–102. 15705329 10.3201/eid1101.040426 PMC3294339

[5] Kaplan JE (1982) Epidemiology of Norwalk gastroenteritis and the role of Norwalk virus in outbreaks of acute nonbacterial gastroenteritis. Annals of Internal Medicine 96, 756–761. 6283977 10.7326/0003-4819-96-6-756

[6] Karst SM (2010) Pathogenesis of noroviruses, emerging RNA viruses. Viruses 2, 748–781. 21994656 10.3390/v2030748 PMC3185648

[7] Lopman BA (2003) Viral gastroenteritis outbreaks in Europe, 1995–2000. Emerging Infectious Diseases 9, 90–96. 12533287 10.3201/eid0901.020184 PMC2873740

[8] Lopman BA (2004) Clinical manifestation of Norovirus gastroenteritis in health care settings. Clinical Infectious Diseases 39, 318–324. 15306997 10.1086/421948

[9] Rockx B (2002) Natural history of human Calicivirus infection: a prospective cohort study. Clinical Infectious Diseases 35, 246–253. 12115089 10.1086/341408

[10] Mounts AW (2000) Cold weather seasonality of gastroenteritis associated with Norwalk-like viruses. Journal of Infectious Diseases 181, 284–287. 10.1086/315586 10804139

[11] Kapikian AZ (1972) Visualization by immune electron microscopy of a 27-nm particle associated with acute infectious nonbacterial gastroenteritis. Journal of Virology 10, 1075–1081. 4117963 10.1128/jvi.10.5.1075-1081.1972 PMC356579

[12] Zahorsky J (1929) Hyperemesis Hiemis or the winter vomiting disease. Archives of Pediatrics & Adolescent Medicine 46, 391–395.

[13] Lopman BA (2003) A summertime peak of “winter vomiting disease”; surveillance of noroviruses in England and Wales, 1995 to 2002. BMC Public Health 3, 13. 12659651 10.1186/1471-2458-3-13 PMC153520

[14] Hall AJ (2011) Incidence of acute gastroenteritis and role of norovirus, Georgia, USA, 2004–2005. Emerging Infectious Diseases 17, 1381–1388. 21801613 10.3201/eid1708.101533 PMC3381564

[15] Moore MD, Goulter RM and Jaykus LA (2015) Human Norovirus as a foodborne pathogen: challenges and developments. Annual Review of Food Science and Technology 6, 411–433. 10.1146/annurev-food-022814-015643 25884284

[16] Tam CC (2012) Longitudinal study of infectious intestinal disease in the UK (IID2 study): incidence in the community and presenting to general practice. Gut 61, 69–77. 21708822 10.1136/gut.2011.238386 PMC3230829

[17] Ashford DA (2003) Planning against biological terrorism: lessons from outbreak investigations. Emerging Infectious Diseases 9, 515–519. 12737732 10.3201/eid0905.020388 PMC2972753

[18] Scallan E (2011) Foodborne illness acquired in the United States – major pathogens. Emerging Infectious Diseases 17, 7–15. 21192848 10.3201/eid1701.P11101 PMC3375761

[19] Statistics Canada (2018) Canada at a Glance 2018 (Population). Available at https://www150.statcan.gc.ca/n1/pub/12-581-x/2018000/pop-eng.htm (Accessed 2 August 2018).

[20] Ontario Ministry of Health and Long-Term Care (2017) Get medical advice: Telehealth Ontario. Available at https://www.ontario.ca/page/get-medical-advice-telehealth-ontario (Accessed 2 August 2018).

[21] Sykes Assistance Services Corporation (2018) Telephone Triage. Available at https://www.sykesassistance.com/services/telephone-triage (Accessed 8 September 2018).

[22] Ministry of Health and Long-Term Care (2011) Teletriage Health Services. 2011 Annual Report of the Office of the Auditor General of Ontario.

[23] van Dijk A (2008) Can Telehealth Ontario respiratory call volume be used as a proxy for emergency department respiratory visit surveillance by public health? Canadian Journal of Emergency Medicine 10, 18–24. 18226314 10.1017/s1481803500009969

[24] Buckingham-Jeffery E (2017) Correcting for day of the week and public holiday effects: improving a national daily syndromic surveillance service for detecting public health threats. BMC Public Health 17, 477. 28525991 10.1186/s12889-017-4372-y PMC5438549

[25] Loveridge P (2010) Vomiting calls to NHS Direct provide an early warning of norovirus outbreaks in hospitals. Journal of Hospital Infection 74, 385–393. 20172625 10.1016/j.jhin.2009.10.007

[26] Pavlin JA (2013) Syndromic surveillance for infectious diseases In M'ikanatha NM, Lynfield R, Van Beneden CA and de Valk H (eds), Infectious Disease Surveillance: Second Edition. Silver Spring: Wiley-Blackwell, pp. 470–481.

[27] Glass RI (2009) Norovirus gastroenteritis. New England Journal of Medicine 361, 1776–1785. 19864676 10.1056/NEJMra0804575 PMC3880795

[28] Best M and Neuhauser D (2006) Walter A Shewhart, 1924, and the Hawthorne factory. Quality and Safety in Health Care 15, 142–143. 16585117 10.1136/qshc.2006.018093 PMC2464836

[29] Wang R (2017) How to select a proper early warning threshold to detect infectious disease outbreaks based on the China infectious disease automated alert and response system (CIDARS). BMC Public Health 17, 570. 28606078 10.1186/s12889-017-4488-0 PMC5468940

[30] Larsson J (2011) Control charts as an early-warning system for workplace health outcomes. Work 39, 409–425. 21811031 10.3233/WOR-2011-1191

[31] van Dijk A (2017) Risk assessment during the Pan American and Parapan American Games, Toronto, 2015. Public Health Reports 132(suppl. 1), 106–110. 28692399 10.1177/0033354917708356 PMC5676513

[32] Siqueira JAM (2015) Application of time series control charts to model and monitor the seasonality of norovirus. Revista Pan-Amazônica de Saúde 6, 61–68.

[33] Tan SSL and Goonawardene N (2017) Internet health information seeking and the patient-physician relationship: a systematic review. Journal of Medical Internet Research 19, e9. 28104579 10.2196/jmir.5729 PMC5290294

[34] Semigran HL (2015) Evaluation of symptom checkers for self diagnosis and triage: audit study. British Medical Journal 351, h3480. 26157077 10.1136/bmj.h3480 PMC4496786

[35] Fox S and Duggan M (2012) Mobile Health 2012. Washington, DC, USA: Pew Research Center.

[36] Trainor E (2013) Detection and molecular characterisation of noroviruses in hospitalised children in Malawi, 1997–2007. Journal of Medical Virology 85, 1299–1306. 23918547 10.1002/jmv.23589 PMC8168456

[37] MacCannell T (2011) Guidelines for the prevention and control of norovirus gastroenteritis outbreaks in healthcare settings. Infection Control and Hospital Epidemiology 32, 939–969. 21931246 10.1086/662025

[38] Bull RA (2006) Emergence of a new norovirus genotype II.4 variant associated with global outbreaks of gastroenteritis. Journal of Clinical Microbiology 44, 327–333. 16455879 10.1128/JCM.44.2.327-333.2006 PMC1392656

[39] Government of New Brunswick (2018) Tele-Care. Available at http://www2.gnb.ca/content/gnb/en/services/services_renderer.8995.Tele-Care.html (Accessed 3 August 2018).

[40] Alberta Health Services (2018) 811 – Health Link. Available at https://www.albertahealthservices.ca/assets/healthinfo/link/index.html (Accessed 3 August 2018).

[41] Triple-S Project (2013) Guidelines for designing and implementing a syndromic surveillance system. French Institute for Public Health Surveillance.

